# Hacking the Predictive Mind †

**DOI:** 10.3390/e26080677

**Published:** 2024-08-10

**Authors:** Andy Clark

**Affiliations:** 1 Department of Philosophy, University of Sussex, Brighton BN1 9RH, UK; andy.clark@sussex.ac.uk; 2 Department of Informatics, University of Sussex, Brighton BN1 9RH, UK

**Keywords:** active inference, computational psychiatry, placebo, pain, functional disorders

## Abstract

According to active inference, constantly running prediction engines in our brain play a large role in delivering all human experience. These predictions help deliver everything we see, hear, touch, and feel. In this paper, I pursue one apparent consequence of this increasingly well-supported view. Given the constant influence of hidden predictions on human experience, can we leverage the power of prediction in the service of human flourishing? Can we learn to hack our own predictive regimes in ways that better serve our needs and purposes? Asking this question rapidly reveals a landscape that is at once familiar and new. It is also challenging, suggesting important questions about scope and dangers while casting further doubt (as if any was needed) on old assumptions about a firm mind/body divide. I review a range of possible hacks, starting with the careful use of placebos, moving on to look at chronic pain and functional disorders, and ending with some speculations concerning the complex role of genetic influences on the predictive brain.

## 1. Preamble: Finding Karl Friston

In July 2009, I was vacationing with my family in the highlands of Scotland. After a long and pleasant pub lunch one day, I was relaxing over a coffee and a glass or two of highland malt when I noticed a short ‘Opinion’ piece by Professor Karl Friston in the journal *Trends in Cognitive Sciences* [1]. It was about the brain as an organ of (multi-level, precision-weighted) prediction, and was entitled “The free-energy principle: a rough guide to the brain?”. The timing was auspicious. I was then half-way through a four-year stint as Head of Philosophy at the University of Edinburgh. I knew I would not get much writing done while in that role and had decided to use what little research time I had to try to improve my knowledge of contemporary computational and cognitive neuroscience. The little paper seemed to fit the bill nicely. In addition, my partner, the cognitive neuroscientist Alexa Morcom, knew Professor Friston from her days at UCL and thought I would enjoy Karl’s signature mix of neuroscientific detail and big-picture theorizing. I devoured the paper in one sitting and was immediately hooked. 

It is no exaggeration to say that reading that article changed the course of my professional life. It did so by providing a plausible neurocomputational foundation for many of the core ideas (about mind, embodiment, and action) that I had been working on for decades. That new foundation is now described in detail in [2]. But even then, I was struck both by how much it seemed to explain and by the elegant and under-stated way Professor Friston managed to expose the deeper (I might even say, ‘philosophical’) importance of the mechanisms under discussion. So much so that I decided to spend the next couple of years simply trying to get to grips with the rich detail of the picture, eventually playing it back to a wider interdisciplinary audience via a well-received target article in the peer-reviewed journal *Behavioral and Brain Sciences* [3]. 

While I was writing (and repeatedly re-writing) that paper, I somehow—encouraged by my partner—summoned up the courage to share a fairly early draft with Professor Friston himself. I remember his response quite vividly. It was friendly, informative, and encouraging—a combination that, I now realize, is classic Karl. My rendition of his (free-energy minimization) view was, he said, ‘almost lyrical’—a lyricism flowing, no doubt, from my obvious inability to follow most of the math! We soon became friends, and I have continued to work on the core ideas ever since, trying to draw them closer and closer together with my own longstanding interests in embodied and situated cognition, revealing brain, body, and world as truly intimate partners in the construction of human experience. The resulting picture reveals predictive brains as the perfect platforms for the embodied minds of active animals [4,5].

In this paper, I make no attempt to rehearse or summarize the key properties of active inference itself. That has been done successfully elsewhere—both by myself [2,4] and by many others, including [6,7]. Instead, and in honour of his 65th birthday, I pursue just a single thread—an opportunity relating to the role of hidden predictions in structuring experience. For those predictions structure not just our experience of the wider world but of our own bodies and medical symptoms too. This delivers some (real but limited) wiggle room for ‘hacking our own predictive minds’—improving our own self-experience by altering some of those hidden layers of prediction. Understanding this principled ‘room for manoeuvre’ may be a major player in the evolution of health care in the 21st century.

## 2. The Power of Placebo

Perhaps the most obvious way to tap into the wiggle room provided by the predictive brain is to harness the power of placebo effects [8]. Placebo effects are health effects brought about by mechanisms including (but not limited to) the patient’s own beliefs and expectancies. They are commonly observed when symptomatic relief is obtained using a ‘clinically inert’ substance that the recipient believes to have active properties. Importantly, as we will see, placebo effects engage specific mechanisms that generate symptomatic relief and should be distinguished from the larger class known as ‘placebo responses’—a class that refers to any condition-related changes observed after the administration of a clinically inactive substance or procedure and may thus include effects such as spontaneous remission or regression to the mean [8].

Contemporary thinking about placebo effects is often traced to a wartime incident in Italy when a physician observed a nurse, responding to a shortage of real pain-killing drugs, administer occasional saline injections instead of a scheduled injection of morphine [9]. In multiple controlled studies, it has been shown that such ‘dose-extending placebo’ regimes work. Repeated administration of the actual drug teaches the brain/body system to ‘expect’ a potent avalanche of pain-relieving responses, many of which can be recreated or approximated by the patient’s own endogenous opioid system. Supporting this picture, placebo relief has been shown to be blocked by drugs that block key opioid receptors. 

The reach into physiology is impressive. Placebo effects in the case of Parkinson’s disease are known to involve effects at the level of dopamine and the basal ganglia. Patients given a saline injection after repeated injections of the anti-Parkinson’s drug apomorphine show apomorphine-like responses visible in neural activity recorded from the ventral anterior and anterior ventrolateral thalamus. In that study, no such responses occurred if the placebo was given without prior experience of the actual drug. But after just four normally spaced apomorphine drug administrations, the response to the placebo was as large as to the real drug [10]. By using this kind of mixed ‘dose-extending’ therapy, it becomes possible to induce precise and effective placebo effects in individuals who start out as non-responders. The same broad profile has been seen for other drugs, including the pain-killing effects of aspirin and ketorolac. 

These are by no means simply superficial effects on what patients say. Instead, placebo effects have been shown to reach far down, altering bodily responses even at the level of the spinal cord [11]. Expectations of relief are actively reaching down, altering the whole cascade of bodily changes and responses from which experience itself is constructed. This may involve actively altering the flow of sensory signals and/or altering the way we sample and attend to such signals [12,13].

‘Placebo analgesia’ whereby an inert preparation causes a reduction in experienced pain is relatively easy to induce and surprisingly effective in many cases. The efficacy varies delicately with how strong we make the expectation of analgesia, e.g., by differently describing the potency and certainty-of-effect of some (actually inert) ingredient. And the higher the estimate of their power, the greater the effect—inert substances delivered by syringe are typically more effective than those delivered by pill [9], presumably because we automatically estimate this as a more powerful form of intervention. Similarly, patients suffering from various forms of knee (and shoulder) pain were found to improve significantly following so-called ‘placebo surgery’, in which the patients were told they have been given an arthroscopic repair but in fact received only sufficient ‘surgery’ to induce a few incision marks on the area [14]. Remarkably, patients receiving the placebo surgery reported similar amounts of relief to those undergoing normal surgery. Such patients also reported more relief than those receiving other, less ‘serious’ placebo interventions, such as pills or coaching, in line with the idea that placebos activate confident hidden predictions that vary with the apparent potency of the intervention. In another kind of case, athletes showed improved performance when hooked up to what they thought were ‘pure oxygen’ delivery systems when in fact they were simply delivering normal air [15]. 

But placebo interventions need not always involve deception. Subtler interventions are possible too. An important range of cases involve the use of ‘honest placebo’. Here, a medically inert substance (or procedure) is given to a subject who knows, with understanding and confidence, that they are receiving an inert substance or a causally ineffectual procedure. In many such cases, benefits were still felt despite the person believing, correctly, that the substance or procedure has no causal powers relevant to the desired effect. High hidden confidence in their efficacy might be triggered simply by details such as interpersonal relations (the manner and sensitivity of the physician), packaging, presentation, or font. The power of the honest placebo was displayed in a 2019 study of patients suffering from cancer-related fatigue (CRF). All subjects were authoritatively informed that the pills they were given contained no active ingredients, and hence ought not be effective in reducing CRF. Nonetheless, the results were striking, leading the authors to conclude that “even when administered openly, placebos improve CRF in cancer survivors” [16]. In an earlier study, an honest placebo was administered to 80 patients suffering from Irritable Bowel Syndrome (IBS), resulting in clinically significant improvements in 59% (against 35% in a control group) [17]. 

The patients taking the honest placebo doubled their rate of improvement, equalling the performance of two prominent (active) IBS medications. In another set of studies published in 2016, 83 patients with chronic lower back pain were assigned to two groups: one group continued their medications as before, while the other group were switched to a clearly presented honest placebo. Based on before-and-after questionnaires, those in the former (no change) group reported a 9% reduction in usual pain, a 16% reduction in maximum pain, and no reduction in disability. But those in the latter (honest placebo) group reported a 30% reduction in both usual and maximum pain and—perhaps most significantly of all for daily purposes—a 29% drop in ‘experienced disability’ [18]. The source of many of these predictions is not belief in the ordinary sense of the word, meaning the sum of the things we might say we predict or expect. Instead, they are the sedimented residue of a lifetime of experience. These predictions are mostly hidden from our own view—but we can still exploit them, thereby hacking our own predictive brains [19].

In addition, recent research suggests that a variety of contextual factors also play a large role in determining experiences of illness and health. These factors include the role of the therapeutic context and the active care, support, and encouragement of the physicians or practitioners. Such factors can help the patient to notice small interoceptive changes that their brain can then take as new evidence for increasingly helpful predictions of ‘incoming health’ [19,20,21]. The take-home message is that potent predictions of improved health can be nudged by a wide variety of placebo-involving and placebo-enhancing means. 

## 3. Limits and Tangles

There is a danger, however, that the emerging science of placebo effects on predictive brains could be hi-jacked by some (by no means all) unscrupulous proponents of alternative therapies. Fabrizio Benedetti, a leading researcher in the field some of whose work we met earlier, warns against the very real danger that hard science could now give an unwelcome (and potentially dangerous) boost to pseudo-science [22]. Placebo hand sanitizers will not kill a virus. In the case of Parkinson’s, placebo drugs reduce pain and muscle rigidity but—and this cannot be over-emphasized—they do not seem in any way to affect the process of neuronal degeneration that underlies the steady progression of the disease. Similarly, no placebo affects the bacteria that cause pneumonia. There is, as Benedetti notes, no evidence that placebo effects occur for many classes of drugs, including anti-platelets and anti-coagulants. Instead, the power of placebo and ritual lies in their power to sculpt the expectations and patterns of attention that construct human experience. Such sculpting involves causal chains of a perfectly respectable kind—as we saw in the case of endogenous opiates. But the progression of disease involves multiple chains and mechanisms, many of which lie far beyond their reach. Benedetti rightly points to education and communication as the keys to getting this right. Understanding how deep-set predictions work with shifting patterns of attention to construct human experience shows why, and how, placebos exert their influence on pain, depression, anxiety, and more. But it should also help us plot the limits of such effects. By better understanding both their scope and limits, placebo effects of many kinds may slowly but surely become incorporated into evidence-led science. 

Appreciating the power of placebos can also lead to personal and ethical tangles. This was brought home to me after my mother was diagnosed with terminal cancer. She was put on a hormone therapy that was briefly very effective in delaying the inevitable, allowing her to celebrate her 80th birthday with customary style and panache. At some point in her treatment, however, the colour of one of the pills that she was taking was altered—from a vibrant pink to a dull blue. My mother insisted that the pink pills made her feel much better. Checking the ingredients, we could find no clinically significant difference. The doctors confirmed as much. My mother firmly but gracefully demurred. But try as we all might, no pink versions could be sourced. 

This put me in a tough spot. I knew that placebo effects were real and potentially helpful against fatigue and anxiety, and I knew that for her, the pink ones really gave her confidence. Yet the best way forward, it seemed to me, was to firmly suppress this thinking on my part and try to convince her of the equal efficacy of the blue pill, thereby (I hoped) rendering it indeed equally efficacious. I also genuinely believed that, in some deep sense, the colour really ought not to matter. Blue really should be as good as pink! And it would be, if only the right set of expectations of relief could be engaged. 

To this day, I am not sure how I should have responded. It was (and remains) a tangle. Notably, it would not have been any different even if I had been the one taking the medication. I think we have a long way to go before a solid picture emerges of how best to leverage potentially quite powerful placebo effects in an honest, helpful, and evidence-led way.

## 4. Ritual, Trust, and Virtual Reality

The general moral is remarkably broad. It is that anything that can (responsibly) be done to increase our confidence in an intervention or procedure is likely to have real benefits. This could simply mean trusting a certain doctor, or hospital, or responding to details of packaging and presentation. In this regard, the active inference framework offers a firm theoretical grounding for emerging work that displays the importance and efficacy of ‘person-centred care’ and the ‘therapeutic alliance’. The core idea here is that by engaging in dyadic or group exchanges, human agents can helpfully alter both the predictions at play and the precision of those predictions. Therapeutic touch, inter-agent synchrony, and verbal interactions can all feature in such therapies [23,24,25,26].

Much that was previously dismissed as ‘mere ritual’ can also fall into place as part and parcel of how to treat human beings whose expectations of pain and relief are themselves an important part of the causal matrix that delivers their own lived experience. These kinds of effects are already (implicitly or explicitly) understood by medical practitioners of many stripes, as well as sports coaches, life coaches, politicians, teachers, advertising companies, sales personnel, and pretty much anyone who ever needs to deal with other human beings. But by locating them as flowing directly from the normal functioning of the predictive brain, we can pave the way for a scientifically well-grounded, evidence-led approach that seeks to understand both the scope and limits of such effects and that explores how best to leverage them.

It is not just pills, potions, and well-targeted procedures that can bring placebo-style relief. Hooking subjects up to soothing music and rich virtual worlds can be potent too. VR provides another promising means to ‘hack the predictive brain’. In a typical experiment in this area, a swarm of pulsing and undulating jellyfish float across the subject’s field of vision. Under these conditions, heat-pad stimulations applied to the subject’s arm were used as a way of testing their ability to cope with increasingly painful intensities. Subjects’ abilities to tolerate higher heat were greatly improved when the heat was applied while immersed in the virtual oceanic world. Unsurprisingly, opioid treatments were robustly successful too. But opioid treatments *combined* with VR resulted in even greater reductions in pain than opioid treatments alone. Neuroimaging results showed altered activity in the VR condition in key neural areas such as the insula and the thalamus, as well as in somatosensory areas [27].

VR treatment of this kind has been used successfully in patients with acute burns, enabling them better to tolerate the changing of wound dressings on the burns, and in patients with phantom limb pain [28,29]. Reductions in experienced pain have also been found in some controlled studies using various forms of music therapy. But here, the results are mixed and often conflicting [30]. Overall, however, there seems to be a potential role for ‘combination’ therapies in which standard treatments (such as the use of opioids) are combined with other forms of intervention, such as the use of VR, to deliver mutually enhanced benefits. 

Why does VR treatment work? The natural, but explanatorily thin, answer is simply ‘by distraction’, by prompting us to attend away from the pain and towards the soothing novelties of the floating jellyfish. But active inference goes further, suggesting both the shape of the underlying mechanism and making sense of the importance of the specific content too. An obvious factor there is the immersive environment. This—as anyone who has tried contemporary VR will attest—is pretty much impossible to ignore. It is alien and surprising—but (being well chosen) never threatening. Such a sensory world acts like a magnet, requiring the brain to scrabble (increasing the weighting on select prediction error signals) to make sense of this new and constantly shifting environment. Increased precision (the relative weighting on predictions and prediction error signals—see [2,4,6]) over this new sensory information means decreased precision (hence decreased influence) for other sensory information, including information regarding the painful procedure. At the same time, the gentle rhythms of the rise and fall of the jellyfish will engage and entrain soothing and helpful bodily predictions (predictions of gently breathing and reduced heart rate, for example). In all these ways, the VR world acts like a kind of prediction template, nudging experience in a better direction. Active inference models thus provide a clearly defined mechanism that explains just why, and to what degree, ‘distraction’ can alter experience itself. By unpacking ‘attention’ as the zero-sum game of variable precision-weighting, they reveal the inner mechanism of ‘distraction’ itself.

## 5. Precision Medicine and Good Expectations

To appreciate both the complexity and the potential of active inference, consider some recent work on improving patient tolerance of statins. These drugs have shown themselves to be a useful part of our on-going medical efforts to combat heart disease, but adherence is poor due in part to a common misperception that the drugs quite often have painful side effects involving muscle pain. The best evidence suggests that although these effects can sometimes occur, they are nowhere near as common as many of us believe. It looks as if a lot of us are simply giving up on statins rather too easily, blaming normal aches and pains on the drugs, and perhaps even developing or amplifying such symptoms purely because we consciously or unconsciously started to predict them [31]. This is another example of the placebo effect working in reverse—the so-called ‘nocebo’ effect, in which expected pain or discomfort acts as another form of self-fulfilling prophecy. 

There is, however, a gene variant that has been shown to be associated with the development of muscle pain and that involves a direct physiological response to certain statin regimens. This allows for a more personalized approach in which genetic screening identifies those at risk and alters the regimen accordingly. This is good news for those with the gene variant, since they can be offered other statins or alternative treatments. 

But being informed of the test results was also found to improve regimen re-instantiation across the board. The best explanation seems to be that simply being told that you are *not* genetically at risk of developing statin-related muscle pain itself helps counteract unhelpful expectations of such pain. This led one of the researchers to comment that “This concept of using precision medicine to address the psychology of how patients feel about drugs might be a winning combination” [32].

In other words, an unexpected benefit of the contemporary move towards precision (i.e., genetically personalized—this is not the technical notion of precision used in active inference) medicine may come directly from the increased confidence we feel when treatments are highly tailored to our own unique situation. This is having a real (clinically standard) treatment and a placebo effect rolled neatly into one. Future humans may benefit both from the better targeting that precision medicine offers, and from any resulting increased confidence in the proposed treatments.

## 6. Does Pain Hurt?

“Pain don’t hurt”—so claimed Dalton (Patrick Swayze) in the 1989 movie Road House. But we all know pain does hurt. When my partner (once a doctor, now a neuroscientist) fell on the slippery steps of our beloved old houseboat, Love and Rockets, the pain was so bad she feared she had cracked a bone in her back. Even once that alarming moment had passed, she could not move off the wet metal stairs until some serious pain meds had kicked in. 

At the same time, we tend to feel that pain certainly can be ‘all in the mind’. If you think you are feeling constant pain in your left toe, then it might well be said that you really are feeling pain, even if there is no physical cause of the usual kinds—no damage to the toe, or to the nerves, no referred pain originating from damage elsewhere. That does not mean there is no physical difference at play. Assuming (as I do) that the mind itself is a material phenomenon, something in that material realm must be different, since you now feel a pain where you felt none before. But the painfulness of our pains can sometimes be remarkably disconnected from even the full matrix of standard gross-bodily or (as they are sometimes misleadingly named) ‘organic’ causes.

Such disconnections, and Dalton’s bald assertion that “pain don’t hurt”, are less puzzling if predictive brains build human perceptual experience by combining expectations (top–down prediction) with bottom–up sensory evidence. If human experience emerges only in this (precision-weighted—see below) combination, then what we might think of as ‘raw sensory evidence’ is never experienced. Experience always and everywhere reflects webs of prior knowledge and expectation.

It is worth saying this again. Sensory evidence, such as the activity at photoreceptors in the eye caused by exposure to a red beetle, is not what we see when we see the red beetle. That seems obvious. But nor is stimulation of the so-called ‘pain receptors’ (called nociceptors) what we feel when we feel a sharp pain. Experience is, in every case, a construct, arising at the meeting point of sensory evidence (stimulations) and own conscious and unconscious expectations. These combine in what amounts to a process of unconscious inference—informed guessing—about the nature and significance of the events causing the sensory stimulations. Sometimes, the end result of that informed guessing is an outward-looking perceptual experience, for example, ‘seeing a cat’, while at other times, it is an inward-looking experience, such as ‘severe pain in my left toe’. But the process is very much the same.

All human experience arises at the meeting point of predictions and sensory stimulations. But how those two forces meet and balance is itself determined by a further factor—one mentioned briefly above. That factor is the brain’s estimate of the reliability (the ‘precision’ or inverse variance) of each—for discussion, see [4,6]. The involvement of both predictions and their precisions is what creates room for manoeuvre on the road leading from raw sensory stimulation to human experience. It is that room for manoeuvre that Dalton appears to be pointing towards—the ‘wiggle room’ that may indeed allow some skilled agents to exert a degree of control over their own pain experiences. That same wiggle room, however, opens up the door to various dysfunctional balances in which pain and other symptoms are constructed in ways deeply dislocated from any standard organic causes. We can feel pains where there is no hidden damage, and we can sometimes feel no pain despite severe damage. Moreover, our own framing and assessment of the significance of our pain matters. Is it a good pain that signifies healing, or a bad pain that signifies ongoing damage and danger? How we experience our own pain can differ significantly as a result of such framing [19].

Active inference provides a deep unifying framework within which to make sense of these and other surprising effects. Understanding a wide spectrum of both normal (‘neurotypical’) and psychologically atypical phenomena in these terms also reveals substantial common ground linking between typical and atypical forms of human experience. This invites us to re-think the age-old picture of a firm mind/body divide and casts doubt on any crude division between ‘psychiatric’ and ‘organic’ causes.

## 7. Beyond Tissue Damage

The picture of pain as a simple response to bodily damage or insult has long been abandoned in both clinical practice and the sciences of mind. A repeated theme in this large literature is that expectations about our own states of pain make a surprisingly large difference to the amounts of pain we experience [33]. 

In work dating back to the 1990s, Professor Irene Tracey and colleagues at the University of Oxford showed that expectations of pain activated key neural circuits relating to experienced painfulness [34]. In one striking fMRI study, they showed that religious beliefs could modulate and regulate the experience of pain, exerting an analgesic effect mediated (they argued) by a kind of high-level re-framing of the sensory signals. When shown religious images, subjects rated a sharp pain as lower (less intense) than atheists shown the same image. But alter the image to one lacking such significance and the pain ratings were equal for both groups [35]

Active inference suggests that such effects depend delicately upon what we are predicting and how we attend to our own bodies—since this influences the reliability (precision) assigned to different predictions. Just as in the case of outward-looking senses such as sight and hearing, the brain clearly does not passively wait for pain information to arrive via the nerves. Instead, it pro-actively predicts the arrival and intensity of nociceptive (‘pain’) information and pro-actively estimates the likely reliability of its own predictions, up- or down-regulating their effects accordingly.

In one fairly typical study, experimenters used heat stimuli to induce different pain intensities while manipulating, using verbal cues, the certainty or uncertainty of the subjects’ expectations about the magnitude of the impending pain [36]. High-intensity painful stimuli were perceived as more painful when their onset was cued with high certainty, while low-intensity painful stimuli were perceived as less painful when cued with high certainty (i.e., under reliable expectation of low pain). But both these effects disappeared when the expectations (here, induced by visual cues) were unreliable—a result that fits well with the idea that estimates of precision determine the balance between top–down prediction and incoming sensory information. Only estimated-as-reliable expectations get to influence the felt sensations in the expected directions, and the higher the estimated reliability, the stronger the effect. 

## 8. Vicious and Virtuous Cycles

Probing and extending this picture, experimenters recently manipulated cue-based expectations of pain in ways that revealed a circular, self-reinforcing nature [37]. During a training phase, participants viewed arbitrary abstract visual cues (geometric shapes). These shapes were consistently paired with pictorial representations of heat intensity (line-drawn images of old-fashioned analogue thermometers showing a lower or higher level of mercury). The idea was to use this training phase to induce differing expectations of heat intensity following the presentation of the abstract (geometric shape) cues. 

Then, in the testing phase, those abstract geometric cues were followed by the application of real ‘noxious contact’ heat stimuli to the inner forearm or lower leg. This was achieved using pads delivering precisely controlled amounts of heat. Unbeknownst to the participants, however, these real heat stimuli were actually exactly the same intensity following all abstract visual cues, regardless of whether the cue was supposed to indicate low or high intensity heat. By keeping the actual intensity of the heat stimulus constant, the experimenters were able to isolate the effects of the cued expectations on experienced pain. Subjects rated both how much pain they expected and how much they felt they received while also undergoing fMRI. 

The brain imaging data were especially useful because they allowed the experimenters to look at the trial-by-trial dynamics not just of expected and experienced pain as it was reported by the subjects but also at the neural activity itself. Specifically, they were looking at the so-called ‘neurologic pain signature’ (NPS). This is a complex neuroimaging (fMRI) signature that had previously been shown to be sensitive to, and specific to, physical pain. The NPS is claimed to act as a kind of window on the basic neural correlates of physical pain, and to be relatively insensitive to various otherwise (more conceptual, symbolic, higher-order) dimensions that might nonetheless affect verbal judgments and responses [38,39]. 

By gathering information about the NPS, the experimenters were able to check, to some degree at least, whether what the subjects said they were experiencing was reflected in the kinds of neural activity that would usually indicate actual physical pain. The results were clear. Both subjective reports and the NPS strongly reflected varying cue-based expectations. As in earlier work, experienced levels of pain were pulled upwards or downwards in the grip of cue-based expectations. But these effects induced future expectations themselves, creating a ‘positive dynamic feedback loop’ in which participants own experiences appeared to confirm their (experimentally misled) expectations, cementing belief in the predictive power of the cues and further contributing to the self-reinforcing cycle. 

Such studies paint a picture of complex dynamics in which expectations, once they get a grip, become increasingly self-confirming and resistant to change. This is a picture that, as we will see, potentially repeats itself in many aspects of human affairs.

## 9. Functional Symptoms

Work on active inference and the predictive brain provides a unifying framework in which we can locate and understand all these kinds of effects, as well as many others including the key role of interoception and the multi-sensory integration of interoceptive, proprioceptive, and exteroceptive signals [40]. In so doing, it positions itself to play a leading role in the newly emerging multi-disciplinary approach known as ‘computational psychiatry’ [41,42]. Computational psychiatry exists at the crossroads between neuroscience and formal computer models of mind. It aims to develop a principled alternative to the standard symptom-based way of classifying and treating mental illness and understanding the variety and scope of normal human experience. Instead of looking at bags of symptoms and focusing directly on associated chemical changes, the new approach seeks to understand mental illness and mental difference as reflections of non-neurotypical computation. Where such an understanding is possible, symptom clusters should begin to make sense, and treatment options (where treatment is appropriate) should become better motivated. The hope is thus to approach mental health and mental illness in the kind of principled and evidence-led way we now approach physical health—by, for example, trying to understand and manipulate the deep causes of ageing and cancer, rather than simply treating the various surface symptoms as they arise.

An especially revealing range of cases, existing at the crossroads of psychiatry and neurology, involves what are now known as functional disorders. These are cases where symptoms such as motor problems, paralysis, or even blindness are present, but no standard physiological cause can be identified. Other names for this include ‘medically unexplained symptoms’, ‘conversion disorders’, ‘psychosomatic’, ‘psychogenic’, or even (in the thankfully quite distant past) ‘hysterical’ disorders. Functional neurological disorders are entirely genuine but appear not to be caused by any kind of anatomical or structural change or conventional disease process. The term ‘functional’ reflects the fact that some aspect of normal functionality (typically involving sensation or movement) is altered or lost despite the apparent absence of any structural or recognized neurological cause—in other words, there is impairment without evidence of systemic damage or disease. Importantly, the presence of a functional disorder is not—and should never be taken to be—evidence of faking or ‘feigning’. Instead, the impairment or disability is real, and there is no implication that it is under deliberate control.

It may be worth pausing for a note on terminology. I will use ‘structural disorder’ and ‘structural damage’ to refer to any cases where there is a standard neurological condition, bodily injury, or disease process present: one whose action already accounts for the experienced pain, disability, or sensory change. I will contrast this with cases of functional pain, disability, or sensory change where similar symptoms are experienced but without any evidence of sufficient, persisting structural causes. In much of the literature, this same distinction is marked using the much more problematic terms ‘organic’ versus ‘functional’—using organic to mean cases where there is some standard neurological condition, bodily injury, or disease process present. I avoid this usage because, to be blunt, it is nonsensical. Functional disorders are as ‘organic’ in origin as any others, and that is in fact one of the most important things that a predictive processing approach can help us to appreciate [43]. 

Functional disorders can be consequences of emotional trauma or stress, but they may also appear in the aftermath of accident or injury if the impairment inexplicably persists long after normal healing processes are complete. They are also frequently (and somewhat confusingly) interleaved with various kinds of impairment and disability whose origins are structural, such as the presence of injury or disease. Nor are they rare. They are the second most common reason (after headache) for new outpatient neurological referrals, accounting for around 16% of all such cases [44]. Functional disorders can present as unexplained cases of blindness, deafness, pain, fatigue, weakness, abnormal gait, tremor, and seizures—in fact, just about any possible impairment [45]. To further complicate matters, many real-world cases present as a puzzling mixture, where physical disease or damage is actually present but is insufficient to account for the degree or variety of pain and incapacity actually experienced. In other words, there are differences in severity of pain or impairment that seem not to be explicable by immediate reference to the underlying cause [46]. 

One perfectly proper response in such cases is, of course, to humbly note that there are often standard underlying causes that have either been missed by the physician or are currently unknown to science. But sometimes, as we will see, the evidence points to a different kind of cause—one involving altered balances within the predictive brain. When this happens, the diagnosis of some form of functional neurological disorder remains a delicate matter. Sufferers will often resist that diagnosis, thinking that they are being told that their very real problem (pain, paralysis, tremor) is in some sense ‘all in their head’. But by better understanding the way all human experience, including medical symptoms with more standard physical causes, is constructed, this kind of stigma may increasingly be avoided. 

What could reasonably lead medical practitioners to diagnose a functional neurological disorder? One striking form of evidence is that the contours of functional problems often follow our intuitive notions of disease or anatomy rather than medically or physiologically sound ones. An example is ‘tubular’ visual field defect. Here, patients with a functional loss of their central visual field often report a visual ‘dead zone’ of exactly the same diameter, no matter how close or far away their visual field is tested. Such a tubular deficit pattern is straightforwardly optically impossible: any visual field deficit must seem to affect more of the visual field when examined at 150 cm from the eye than it does when tested at 50 cm from the eye. For example, if you hold this book up 50 cm from your face, it will appear to occupy more of your visual field than it would if viewed from 150 cm. Tubular visual field defects do not conform to this optically inevitable pattern. This suggests that the defect is functional in nature: there is a genuine loss of visual function, a blind zone, that simply cannot (given the laws of optics) reflect underlying damage to the visual system itself [47]. 

The important point to appreciate is not that the pattern of impairment here is optically impossible (though that is a solid clue that there is something unusual going on). Rather, it is that the shape of the impairment follows the shape of the person’s own expectations and predictions. Their brain strongly predicts a uniform tunnel-shaped loss of vision and that is what they then experience. That kind of uniform-diameter loss is not optically possible and could not be caused by any form of structural damage to the visual pathways or visual processing areas themselves. It could, however, be caused by the person’s own hidden predictions about their own visual experience. Even so, there is no implication—and again, I cannot stress this too strongly—that the person with tubular visual field loss is deliberately predicting that loss, and thus feigning, or intentionally causing, their own disability. The blind region is 100% real and the experience of blindness is involuntary. But its shape reflects the way our own hidden beliefs and expectations are sculpting our experience.

Another—even more dramatic—example comes from University of Edinburgh neurologist Professor Jon Stone. Stone recounts the tale of a teenager whose vision progressively worsened until one day she woke up effectively blind [48]. Extensive tests revealed nothing structurally amiss with her eyes or brain. She was diagnosed as having a functional neurological disorder. In the past, it was thought that such disorders were always due to abuse, stress, or trauma. Nowadays, it is clear that this is not the case. Abuse or trauma can be precipitating factors, but so can physical injury, other forms of disease, or sometimes nothing (nothing obvious at any rate) at all. In the case of the blind teenager, it turned out that she had a history of migraines and that these were triggered by light. As a result, she spent long periods in dark rooms.

Stone suspected that the teenager’s aversion to light and her increasing experiences of darkness had somehow tricked her brain into constantly predicting darkness, and that this lay behind her functional blindness. To push back against those hidden predictions, he showed her that her brain was still receiving good sensory evidence via her eyes. He did this by, for example, pointing out that she was often making eye contact with him or copying his gestures—despite none of that making it into her conscious awareness. He also used a technique known as TMS (Transcranial Magnetic Stimulation), which uses a magnetic field to induce activity in neurons in the brain. Carefully applied, this causes the visual centres to fire. Under those conditions, she ‘saw’ phosphenes (flashes of light) and this, Stone conjectures, may have helped her brain to learn that the predictions of ‘not seeing anything’ were misguided.

With these interventions, and with a lot of careful scene-setting in which Stone and colleagues explained the nature and possible origins of functional disorders, the teenager regained her sight, eventually making a full recovery [48]. Of course, it is impossible to prove that the recovery was a direct result of the interventions. But Stone documents other such cases, in which recovery occurs following similar patterns of explanation and intervention. Predictive processing offers a compelling general picture that makes sense of both the existence of functional disorders, and the apparent efficacy of these forms of treatment. At the core of that picture is the idea that predictions about our own sensory capacities or physical abilities are playing a key role, causing genuinely experienced symptoms to fall into line with those hidden expectations. 

## 10. The Role of Attention

Why, in these cases, do misplaced predictions and expectations get to play such a strong role, sometimes carving experiences out of whole cloth? The explanation for this seems to lie with hidden disturbances to the brain’s mechanisms for estimating precision—mechanisms that, thereby (active inference suggests), deliver attention in all its forms. Aberrant precision estimations are now thought to be implicated in a wide range of psychiatric and functional disorders, as well as in determining the range and variety of more neurotypical response. 

Recall that precision, in these models, is simply a weighting factor that can amplify and dampen different aspects of processing. In brains, precision-weighting involves the action of (among other things) complex neurotransmitter systems centred on dopamine and other chemical messengers. Their co-ordinated action amplifies some aspects of neuronal activity at the expense of others [49]. Varying estimates of precision alter patterns of post-synaptic influence and so determine what (right here, right now) to rely on and what to ignore. This is also the way brains balance the influence of sensory evidence against predictions. In other words, precision variations control which bits of what we know and what we sense will be most influential, moment-by-moment, in bringing about further processing and actions. Expressed like that, the intimacy of precision and attention is apparent. 

What happens when precision estimations misfire? This would skew the impact of different bits of sensory evidence, and of different predictions. This is exactly what seems to be happening with functional disorders. In these cases, unwilled misallocations of precision seed self-fulfilling prophecies. Predictions of pain or impairment become highly over-weighted, and those predictions overwhelm the actual sensory evidence, forcing experience to conform to (and thereby seem to confirm) our own hidden expectations.

There is good evidence that misfiring precision assignments (unusual patterns of attention) play a role in many, perhaps all, functional neurological disorders. For example, simply distracting the sufferer by making them direct their attention elsewhere often makes functional (but not structural) tremors vanish. Patients with these tremors also spend much longer looking at them than those whose tremors have standard causes and they greatly overestimate how often their tremor occurs [50]. When tremors are caused by structural (i.e., standard neurological) disorders, patients’ estimates are much closer to the true frequency. This makes sense if it is disordered attention that, in the case of functional disorder, drives the formation of the tremor itself. In these cases, the process of attending to (‘selectively sampling’ [51]) the tremor ups the precision-weighting on the hidden expectation of tremor and this brings the tremor about. 

## 11. Hoover’s Sign

A classic demonstration of the role of aberrant attention and selective sampling in functional disorders involves the phenomenon known as ‘Hoover’s sign’. Named after the American physician Charles Franklin Hoover, this is present when a patient with unexplained weakness in one leg proves able, when their attention is directed elsewhere, to exert normal amounts of pressure with that leg. The patient is asked to make a certain movement with their non-afflicted leg while the examining doctor checks for pressure exerted on the other (afflicted) side. In normally functioning individuals, there is a kind of crossover effect, such that lifting (say) the left leg causes the opposing hip to extend and the heel of the right foot to exert downward pressure. In cases of structural right leg weakness, this crossover effect is missing, as the right leg cannot respond. But if the weakness is functional in nature, the patient will involuntarily engage the afflicted leg, exerting downward pressure with the heel. The doctor’s request diverts attention to the unafflicted limb, thereby revealing the preserved bio-mechanical ability of the afflicted one [47]. 

Explaining this to patients, the neurologist Jon Stone likes to emphasize the difference between their voluntary leg movement, which is severely impaired, and their involuntary (automatic) movements, which are not. What Hoover’s sign shows is that the problem is not really with the power of the leg, nor even with the ability of their brain, when distracted, to deploy that power. Rather, it reflects what happens when attention is directed towards using the afflicted leg. This is a clever test, and it is widely used today.

The early (circa 1908) literature on Hoover’s sign depicted it as a means of detecting both ‘malingering’ and functional leg weakness [52]. But nowadays, and especially given the picture emerging from predictive processing, there is no implication that the patient is in any way ‘faking it’. Rather, what Hoover’s sign suggests is that a certain unwilled pattern of expectation and attention—caused, of course, by very real changes somewhere within the brain—may be the hidden cause of the apparent weakness. Another way to think about this is that the absence of disease or gross physical damage does not mean that there is no pathological change at all. But the relevant changes are subtle and deep—they reflect a fault in the complex patterns of signal amplification and dampening that are always occurring within the predictive brain. Strongly anticipating pain, numbness, weakness, or other symptoms alters patterns of attention (precision-weightings) in ways that can amplify or entirely generate the experience—which then seems to confirm those very expectations. 

## 12. Expectancy and Its Role in Chronic Pain and Other Conditions

Functional disorders afford a powerful illustration of the role of hidden predictions and patterns of attention. Since all human experience is constructed from mixtures of expectation, attention, and sensory stimulation, it will never be possible to experience either the world or your own body ‘as it really is’. Indeed, it rapidly becomes unclear what this could even mean. Instead, there exists a deep continuity between cases where expectation and attention create symptoms (as mentioned earlier) ’from whole cloth’ and cases where they also reflect the operation of some more standard form of disease or injury. Functional disorders simply lie at one end of this spectrum. 

There is plenty of evidence for this initially surprising claim. Omer Van den Bergh, a health psychologist working at KU Leuven University in the Netherlands, notes that symptoms across a wide range of conditions strongly match their bodily causes only for early, acute, and localized dysfunction or pain—for example, the temporary sharp pains caused by surgery, cuts, and broken bones. Move to chronic conditions and the picture looks very different. For example, reported breathlessness in chronic obstructive pulmonary disease shows huge variation for the same level of lung damage, both in different patients and in the same patients at different times. Similar results were found in studies involving reports of atrial fibrillation, asthma symptoms, diabetes, and many more [46,53]. For example, asthma patients can often experience symptoms in a way that does not reflect their current pulmonary state but is instead the result of ingrained expectations [54]. Typically, such expectancy-driven attacks—variously estimated to affect somewhere between 15 and 60% of sufferers—occur when returning to a context (or encountering a cue) that was associated with a previous attack. This rapidly sets up another self-confirming cycle in which the new attack, being again experienced in that context, seems to confirm the expectation, making such attacks more likely in the future. This is rather like the case of the performer with stage fright whose true abilities are masked by their own mounting expectations of failure. The circularity is daunting. Every new instance of stage fright confirms the (conscious or hidden) expectations of failure, and those expectations ensure that the instances of failure accumulate. Recognizing this circularity is, however, often the first step in breaking the cycle. For a full account of the key role of hidden expectations in many such cases, see [53].

Something similar seems to be occurring in some cases of chronic back pain. In a 2019 interview [55], the London-based health psychologist Tamar Pincus commented that “after several bouts of back pain, people start to process the world differently…their pain becomes embedded [among] the things they associate with themselves. If they are shown an image of a staircase, for instance, their first thought is, “I can’t climb it”. After a while, you see and feel things coated with pain. You no longer need the injury to feel pain. And you might experience more intense pain, purely because you’re expecting it”.

Individuals will differ in how they assign precision to bodily signals, including those associated with pain and disability. But living with a condition for a long time enables idiosyncratic expectations (for example, about severity in different contexts) to arise and become ingrained. This means that even where there is some standard structural cause present (such as a bulging or herniated disc in someone with back pain) the way we experience our symptoms may over time come to involve large doses of mind-set and expectation. 

In a certain sense, chronic pain at that point is perhaps best considered not so much as a symptom, but as the disease itself—the very state that needed to be addressed [56]. On the first Global Day Against Pain in 2004, it was declared that “chronic and recurrent pain is a specific health care problem, a disease in its own right.” Since then, this once-marginal viewpoint has become increasingly influential in both theory and in clinical practice. Active inference provides a powerful new theoretical framework within which this strong claim can be defended and made precise. 

## 13. Pain Reprocessing Theory

With this picture of pain on the table, consider a promising (though still in its infancy) approach known as Pain Reprocessing Theory. The starting point here is the observation that the basic experience of pain seems to be linked to a prediction that our bodies are in imminent or immediate danger—so that if we do not stop whatever it is we are doing, the result will be further nasty bodily damage. This is true enough in the case of, say, a newly fractured ankle. But in many cases of chronic (long-term) pain and disability, a locked-in prediction of imminent further damage seems itself to have become part of the problem. Experienced pain in these cases is (as is often said) a bit like a malfunctioning warning light in a car. Flashing red, it looks like it signals bad trouble—better stop and get off that road quick. But suppose it is the warning light itself that is malfunctioning? 

A core example (one that we mentioned earlier) is chronic back pain. In around 85% of cases, no sufficient ‘standard cause’ (peripheral etiology) can be identified. Pain Reprocessing Theory (PRT) seeks to push back against any misfiring warning light by re-framing the pain in ways that gently unseat those malfunctioning predictions [57]. Elements of the approach include teaching patients about the way chronic pain can sometimes involve a kind of false alarm rooted in locked-in predictions, and then, crucially, slowly installing different predictions—mostly by encouraging sufferers to do a bit more stuff despite the pain. It is almost as if, just by keeping driving that car, you could slowly make the warning light become dimmer. 

Perhaps the single most crucial element in bringing about positive outcomes in such cases is explaining to an affected individual that their experienced pain or fatigue, although 100% real, need not signify any threat of imminent damage and that their felt inability to perform certain tasks is thus acting more like a cause of disability than a reflection of it. A more detailed version of that kind of re-framing could include describing exactly how it is that genuine pain can be caused by misfiring prediction systems. Patients may also be advised to attend to the sensations in as much detail as they can, but not under the label ‘pain’ or ‘hurt’. All this is an attempt to destabilize the role of aberrant attending (aberrant precision weightings) and misfiring predictions, so as to unseat the old inference from pain to incapacity, allowing the formation of a new and more helpful set of self-expectations. Such approaches fall into place alongside an important body of evidence showing that chronic pain conditions frequently involve aberrant perceptions of the affected site, so that correcting these perceptions becomes a key component of any effective treatment [58,59]. In the light of all this, physicians can now stress that chronic pain is curable—and this itself then sets up a new high-level expectation that can again play a powerful role in bringing about positive change. The overall effect (over time) is to initiate a helpful cycle of altered hidden expectations that can lead to large reductions in experienced pain. 

There is some solid emerging evidence (in the form of randomized clinical trials) for the efficacy of PRT [60,61]. These studies found large and sustained reductions in pain and disability following PRT (as compared with both placebo and usual care) in 73% of cases. But it should be stressed that such evidence, although promising, remains preliminary. Moreover, it will be essential to compare PRT with other approaches—such as cognitive functional therapy—that differ in details and emphasis but that likewise show promise for leveraging the power of prediction in the service of relief [62,63].

## 14. Genes for ‘Phenomenological Control’?

We have explored some of the many ways that we can hack our own predictive mind. Intriguingly, these hacks work better for some people than others. Emerging work tracks significant individual differences in the extent to which different individuals can benefit from such effects. This work suggests that some individuals are experts at ‘phenomenological control’—the capacity to exert a kind of unconscious control over the shape of their own experience [64,65].

Some of those differences will themselves be the result of varying life histories, environments, and practices—how well, or badly, we have learnt to nudge our own predictive minds. But one neglected factor turns out to be genetic variation. There is suggestive evidence just emerging that genetic differences in dopaminergic neurotransmission play at least some role in determining individual variations in the control of experience by multi-level predictions. Understanding this requires a small diversion into neurophysiology, but it rewards the effort. 

One key player looks to be the enzyme COMT (Catechol-O-methyltransferase). COMT (acting with other enzymes) helps determine the levels of dopamine in key brain areas. It does this by helping metabolize dopamine, especially in the prefrontal cortex, where it is responsible for degrading more than 60% of dopamine. Genetic variants (differences in DNA sequence) can either enhance or dampen such effects, leading to higher or lower levels of bioavailable dopamine [66]. 

Recall that dopamine is a key player in enabling the complex precision-weighting balancing act that determines which predictions, and which pieces of sensory evidence, have the greatest influence at any given moment. For this reason, increased stocks of dopamine might well result in potentially stronger influences of expectation. Brains with more available dopamine would be able to exert more control over experience. This would be a very exciting discovery, directly linking differences in the efficacy of our own predictions to differences in key resources thought to implement predictive processing in the brain. 

There is reason to think such a link is real. Work by the Harvard Medical School molecular biologist Kathryn Hall and her colleagues has shown that COMT levels (which vary between individuals) correlate well with differing levels of placebo responsiveness. Those with higher levels of COMT (who metabolize more dopamine, thereby reducing its bioavailability) exhibit weaker placebo response. Lower levels of COMT have the opposite effect, seeming to promote placebo responsivity [67,68]. 

Genetic variation of this kind has also been implicated in differing tendencies to form strong beliefs on the basis of scant evidence and may play some role in explaining different mental styles and patterns of creative thought [69,70]. Excessive dopaminergic signalling has also long been thought to play a role in the development of conditions such as schizophrenia [71]. All this falls into place if genetically influenced individual variation alters the patterns of precision-weighted information flow within the predictive brain.

## 15. Conclusions

We are catching a glimpse of something that will, I suspect, prove to be of immeasurable importance in the near-future unfolding of our self-understanding as a species. That something is the shape and nature of the tangled web of interactions and influence linking brain-based predictions, genetic variation, our own life history and practices, pharmacological interventions, environmental factors and forces, and the moment-by-moment unfolding of human experience. Work on active inference provides the first framework with the power to link and triangulate these heterogenous factors and forces in a truly principled way. 

Nonetheless, the picture remains importantly incomplete. There is interesting active inference work on the nature of conscious experience, but it remains speculative and incomplete—see, e.g., [72,73,74]. A major shortfall concerns exactly how our conscious (often linguistically formulated) expectations interact with all those hidden wells of non-conscious prediction. There is no doubt that they do. We saw, for example, that manipulable conscious expectations make a systematic difference to experienced pain. But—as the work on honest placebos so neatly demonstrates—there are layer upon layer of non-conscious predictions also in play. We not yet understand these key interactions, or exactly why it is that explicit conscious predictions can sometimes play greater or lesser roles in sculpting experience. Future work should target the detailed nature of this influence. Improving our understanding of this process is essential if we are to become systematic and effective ‘hackers’ of our own predictive brains. 

On the horizon there glimmers a new evidence-based science—one linking the ebb and flow of predictions and precision-weighted error signals to all the many large and subtle variations in how we experience and act upon our worlds. With that science in hand, interventions upon experience should become much more reliable and targeted. As we become more and more comfortable with this new science, we may finally grasp the fundamental unity of mind, body, and world.

## Data Availability

The original contributions presented in the study are included in the article, further inquiries can be directed to the corresponding author.
